# Optimal SoC range determination for battery storage to smooth wind power output and extend battery lifespan

**DOI:** 10.1371/journal.pone.0333597

**Published:** 2025-10-24

**Authors:** Mehran Alitabar, Mohsen Sedighi, Seyed Mehdi Abedi Pahnehkolaei, Alireza Ghafouri

**Affiliations:** Department of Electrical Engineering, Sar.C., Islamic Azad University, Sari, Iran; Aalto University, FINLAND

## Abstract

The increase in the consumption of electrical energy in the world has increased the trend towards renewable energy for the production of electricity in small and large scales. One of the major renewable energies that have attracted the attention of experts are wind turbine (WT) resources. Due to their dependence on wind speed, these sources have large fluctuations in output power. For this purpose, it is necessary to use electrical energy storage devices that can reduce the fluctuations of wind turbine output power by proper and fast charging and discharging. The use of such energy storage systems also increases network costs and operational complexity. Also, considering that wind turbine output power fluctuations are high and at a high speed, the charging and discharging of these energy storage devices will also occur with a large number of times and will lead to a reduction in the life of this equipment. If the size of these batteries is chosen in such a way that they can be charged and discharged in smaller intervals, their lifespan will be improved and the use of these equipment will be in more favorable conditions. In this paper, an attempt will be made to choose the appropriate state of charge (SoC) range for energy storage devices along with wind turbine resources. The simulation of wind turbine and battery storage in micro-grid and in on-grid condition has been implemented in MATLAB software.

## 1. Introduction

The use of wind energy in the production of electrical energy on a large scale is not a choice but a compulsion in the path of diversification of electrical energy production due to its various economic and environmental advantages. Among the different types of renewable energy, wind energy, along with solar energy, is one of the most widely used types of electrical energy [1]. Many countries around the world are trying to increase the use of this energy as much as possible and increase its share in their electricity production. This progress in energy production through wind power plants are still increasing [2].

Along with all the advantages of developing and using wind energy, the main challenge in this regard is the low energy density and extreme fluctuations in the output power of wind units. Wind energy production sources generally have large fluctuations, which is a matter of uncertainty enters the electrical energy production system, and the result of this is the strong need for reserve rotation or more fast response power plants. This increases the cost of electricity production. On this basis, in order to improve and reduce uncertainty in wind energy production, it is necessary to make accurate forecasts of wind production in order to reduce these costs [3].

Furthermore, robust energy storage planning methods have also been explored to address wind uncertainty and extreme events, such as in [4], where a novel ESS planning approach is proposed to simultaneously reduce wind curtailment and load shedding by modeling spatio-temporal uncertainties during hurricanes and applying an improved column-and-constraint generation algorithm.

Based on this, in [5] a review on reducing the fluctuations of the output power of wind sources and improving its performance with the presence and without the presence of the energy storage system. Reference [6] also determines the energy storage size connected to wind sources have been discussed.

References [7–9] review on the control of wind resources and energy storage system to reduce power output fluctuations and have given suggestions to improve the power output. Reference [10] has also done the same procedure for energy management. In these articles, only the reduction of fluctuation has been discussed and the impact of energy reduction has not been considered. Also, due to the inertia of wind sources, there is still fluctuation in the output power.

In [11], two control models are proposed to reduce the fluctuations of wind turbine output power and compared with each other. These two models include the model TSR (Tip Speed Ratio) and OTC (Optimum Torque Control) may to be. This paper focuses only on MPPT for synchronous generator wind turbines and does not consider the presence of an energy storage system. While wind energy always comes with energy storage system. Reference [12] also reduces the fluctuation of output power for a PMSG wind turbine.

Reference [13] also uses the fluctuations of the power output of wind turbine. The methodology of this paper is control of wind turbine by inertia. The shortcoming of this article is not considering energy storage system.

Reference [14] investigates power output fluctuation mitigation for wind sources connected to weak networks and proposes improvements in that context. This article considers the group of wind farm next to a weak network or the micro-grid, and naturally, the power output fluctuation of the wind farm is less than that of a wind turbine. It should also be noted that in some micro-grid configurations, only a single wind turbine may be utilized as the primary renewable energy source. Therefore, this shortcoming needs to be compensated.

In [15], using wind speed prediction and based on the monitoring control unit and energy storage, the power output has been smoothed. In this reference energy storage and the reduction of fluctuation has been considered. However, the reduction of volatility is not acceptable. Also, the amount of energy reduction obtained from the control is not taken into account.

Reference [16] also shows the performance of the eternal turbine and its output power by controlling the blade angle (Pitch) and improved it using the proposed method. Also in this reference, due to the fact that only blade angle control has been used, the reduction of the power level is evident.

Reference [17] also controls wind turbine power output fluctuations based on artificial neural network (ANN) and capacitor. In this reference, the effect of the network on the wind resource is not considered, and only the effect of the wind turbine is considered.

Reference [18] also suggested to improve wind turbine generator and battery storage in unbalanced radial distribution network have given. Obviously, this procedure is a special state of the network and if the state of the network changes, the output results will not be available.

Reference [19] energy storage size to connect to the wind source and achieve maximum energy in the presence of the system (photovoltaic system). In [20], from fuel cell units, wind turbine, photovoltaic system and energy storage maker as well as interest has used the predictive model to sell electricity. Also reference [21] with the proposal of a complete model and with a ultra-capacitor device has reduced the fluctuations of the power output of the wind source. Control converter between wind source and ultra-capacitor device is designed based on the fuzzy algorithm. In these references, the combined system of several sources is included, which is the strength of these references compared to previous works. However, the state of extracted energy is not taken into account.

Reference [22] and [23] has reduced the fluctuation of wind turbine output power by using wind speed prediction. Reference [24] has also increased the energy obtained from wind turbine sources by using wind speed forecast.

Based on [25] and according to fuzzy algorithm, the fluctuation of the output power in the wind turbine has been reduced. Also, according to [26], by using Li-Ion batteries, the fluctuation of wind turbine output power has been reduced.

Also, as we know, the lifespan of electrical network equipment is very important. How to use energy storage batteries and peripheral equipment will affect the lifespan of these parts. References [27–29] have proposed a procedure to determine the aging of electrical network equipment.

According to the contents and literature review, Table 1 shows the aspects, research gaps of past works and innovations expressed in the relevant references as well as the present paper. According to this table, improving the use of battery energy storage with the aim of improving the power fluctuation and energy harvested from the wind turbine is the most important goals and innovations of this article, which are going to be stated below.

**Table 1:** Compare the proposed method and previous methods.

No.	Aspect	[6]	[7]	[8]	[18]	[19]	[20]	[21]	[22]	[23]	[24]	[26]	This paper
**1**	**WT Power Smoothing**		**✓**	**✓**				**✓**	**✓**		**✓**	**✓**	**✓**
**2**	**Size of ESS**	**✓**		**✓**	**✓**	**✓**					**✓**		**✓**
**3**	**Improving Energy Exchanged**					**✓**	**✓**		**✓**	**✓**			**✓**
**4**	**Prediction Model**						**✓**			**✓**			
**5**	**Improving ESS Operation**												**✓**

WT: Wind Turbine | ESS: Energy Storage System.

The rest of this paper is constructed as follows. In section 2, wind turbine, battery capacity and micro-grid connection modelling are described. In section 3, proposed procedure statement including roughness and smoothing index and proper battery usage scenarios are presented. Next, in the section 4, the results of the simulations of the proposed procedure are stated, and then in the section 5, the results obtained from different scenarios are discussed. Eventually the paper concludes in Section 6.

## 2. Modeling

In this section, the modeling considered in the article will be discussed. Accordingly, the mathematical model of the wind turbine, including its pitch control system, is first described, then the battery capacity and the micro-grid connection modeling are described.

### 2.1. Wind turbine modeling

The power output from the wind turbine (P_t_) according to (1) is obtained [22–23]:


$$P_{t}=\frac{\text{1}}{\text{2}}\rho AC_{p}\left(\lambda ,\theta \right)v^{\text{3}}$$ (1)

In (1), ρ is equal to the air density in kg/m^3^, A is equal to the area swept by the rotor blades in m^2^, v is equal to the wind speed in m/s and C_P_ is the power factor of the turbine. It should be noted that this power factor is a function of the blade tip speed ratio (TSR or λ) and pitch angle (θ). The value of TSR is expressed according to (2):


$$\lambda =\frac{R\omega_{t}}{V}$$ (2)

So that ω_t_ is the rotor speed of the turbine. C_P_ is also a non-linear function of wind turbine components and loss values in the energy conversion process. This value is approximated using (3).


$$C_{p}\left(\lambda ,\theta \right)=0.5176\left(\frac{116}{k}-0.4\theta -5\right)e^{\frac{-21}{k}}+0.0068\lambda$$ (3)

So that:


$$\frac{\text{1}}{k}=\frac{\text{1}}{\lambda +0.08\theta }-\frac{0.035}{\theta^{\text{3}}+1}$$ (4)

For the pitch angle of 0 degrees, the maximum value of the power factor can be obtained, which is in the blade tip speed ratio (TSR) is equal to 7.96, this power factor is equal to 0.411. As the value of the blade angle increases, the range of blade tip speed ratio becomes wider and the maximum value of C_P_ decreases.

### 2.2. Battery energy storage system (BESS) capacity modeling

**Fig 1:**
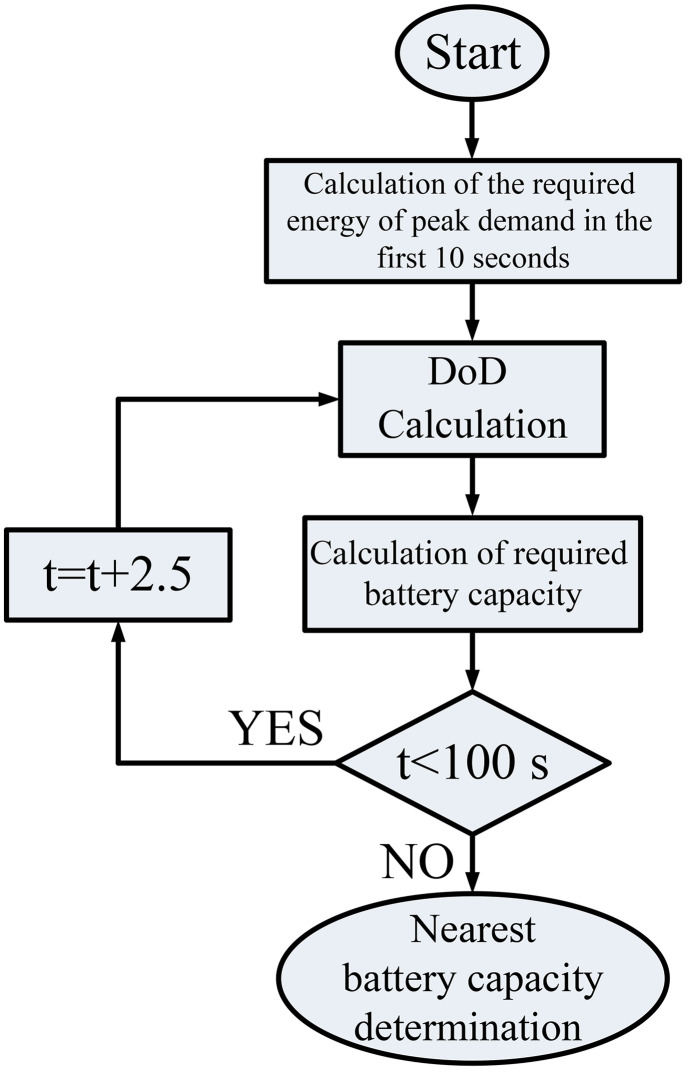
Battery capacity determination procedure.

To determine the battery capacity, the proposed procedure according to Fig 1 will be used.

According to this procedure, the following steps will be followed in order:

- At first, the amount of energy needed for peak demand in the first 10 seconds of the micro-grid is calculated. This value will be calculated as (5):

$$\text{Required\textasciitilde{}Energy}=P_{Peak}\times time$$ (5)

- Then, a value for the Depth of Discharge (DoD) of the battery is determined according to the specifications of the manufacturer. A typical DoD for BESS is around 50%, while lithium-ion batteries can be as low as 20%.
- Next, the battery capacity will be obtained according to (2):

$$\text{BC}=\frac{\text{Required\textasciitilde{}Energy}}{DoD\times \eta }$$ (6)

where BC is battery capacity. In the above relationship, the efficiency value of the battery is desired.

- This procedure is followed for the entire simulation period and the proposed procedure will be repeated at each stage according to the amount of energy required related to the load of the micro-grid.
- At the end, the closest battery capacity available in the market will be proposed for the project. It should be noted that this capacity should be greater than the obtained capacity in order to ensure energy storage during the peak demand period.

### 2.3. Micro-grid connection modeling

**Fig 2:**
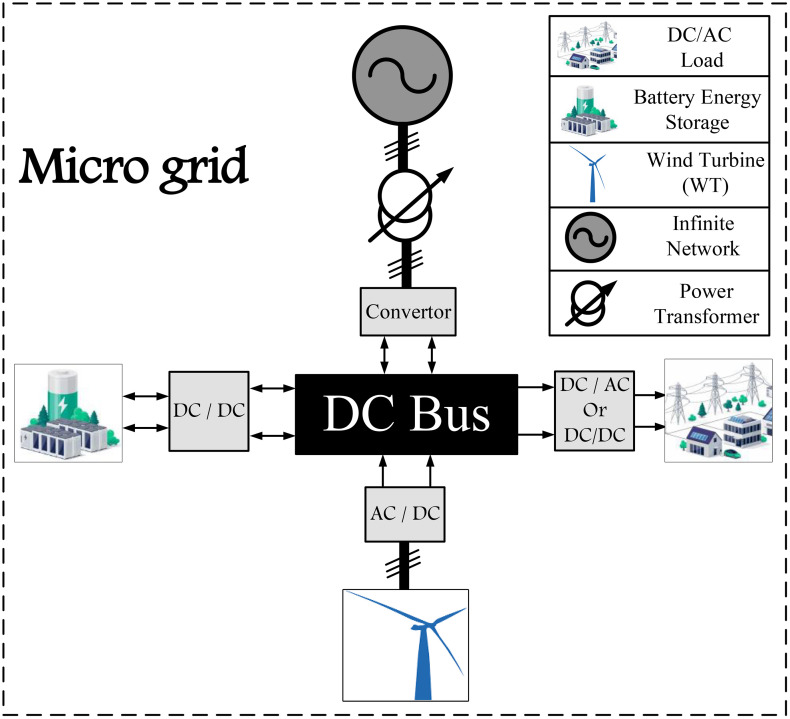
Structure connection of wind turbine, battery and micro-grid.

Various designs have been considered to connect the wind turbine and the battery storage system. In this article, the energy storage system is considered in parallel with the wind turbine system. This wind turbine and battery storage are connected to a weak grid. This type of design is used in reference [22]. The schematic design of this type of connection is shown in Fig 2. The desired loads can be of both types of AC and DC loads.

## 3. Proposed procedure statement

**Fig 3:**
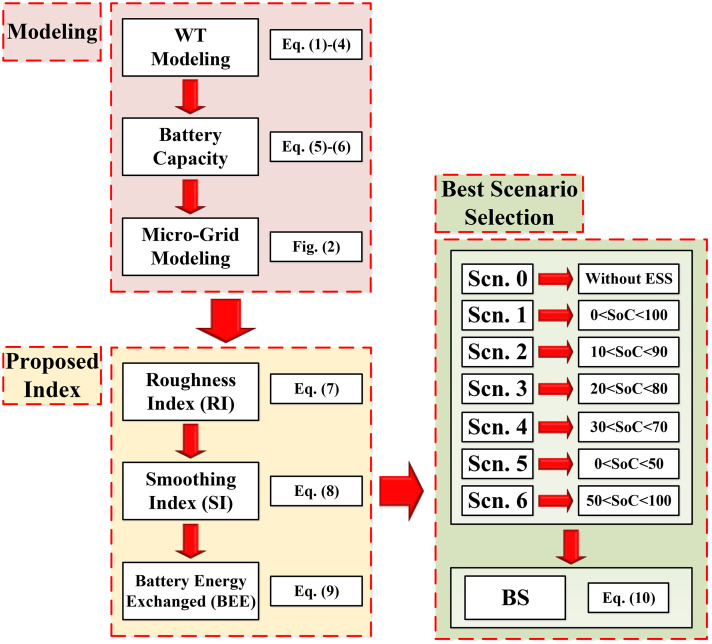
Flowchart of proposed procedure.

In this section, the proposed method of the article will be described. The flowchart of proposed procedure in this paper is shown in Fig 3. According to this procedure,

- At first, the wind turbine, the amount of battery capacity and also the desired micro-grid will be modeled.
- So, the roughness index (RI) and smoothing index (SI) are introduced, and then, using the introduced index and the battery size, battery energy exchanged (BEE) will be evaluated.
- Finally, by introducing different scenarios, the best scenario (BS) will be suggested for the operation of battery storage next to the wind turbine.

The modeling of wind turbine, battery capacity and intended micro-grid are described in section 2. In the rest of this section, proposed index and proper battery usage scenarios will be described in section 3.1 and 3.2, respectively.

### 3.1. Roughness and smoothing index

The roughness index will be in (7):


$$RI=\sum_{n=1}^{n_{spikes}}\left|P_{n}-P_{ave}\right|+\sum_{n=1}^{n_{valleys}}\left|P_{n}-P_{ave}\right|$$ (7)

where P_n_ is the power of the peaks and valleys of the output power curve and P_ave_ represents the average output power. Also, the n_spikes_ and n_valleys_ parameters show the number of output power peaks and valleys, which are actually the local maxima and minimums of the output power signal. The RI index is also the roughness index of output power. According to this relationship, the higher the RI roughness index, the higher the roughness of the output power, and in other words, the more output power fluctuates. It should be noted that the index of this index is in watts.

So, the smoothing index can be proposed as (8):


$$SI=\left(1-\frac{RI_{i}}{\max(RI_{i})}\right)\times 100\%\;\;\;\;,\;i=number\;of\;scenario$$ (8)

In the above relationship, SI is the smoothing index or uniformity index and RI values are the roughness index for different scenarios. The value of max(RI_i_) is also expressed with the aim of unitization the roughness index. It is also important to mention that this index has no unit and is expressed as a percentage. The closer this index to zero indicates a worse production capacity. In other words, the larger this unit and the closer it is to 100%, the better and smoother the state of production power.

Also, the amount of exchanged energy by the battery during the simulation period will be expressed as (9). According to this equation, the exchanged energy in the battery includes the state of charging and discharging, and in both states, the battery is in the circuit and participates in improving the smoother power of the WT.


$$BEE=\sum_{t=0}^{T}\left|BP_{t}\right|$$ (9)

where BEE is the battery energy exchanged, T is the simulation period and BP is the battery power exchanged during the simulation period.

### 3.2. Proper battery usage scenarios

In order to analyze the proper use of the battery, it is necessary to design scenarios for the operating range of charging and discharging the battery. In relation to how to use the battery, the following points should be considered:

- The shorter the battery SoC changes, the longer the battery life will be.
- The smaller the range of SoC changes, the higher the fluctuation of the output power of the wind turbine and battery storage.
- Therefore, deriving a model that fluctuates in a small SoC range and also has small output power fluctuations at the same time will be very practical.

Based on this, the best scenario among the battery storage system exploitation scenarios will be expressed according to (10).


$$BS=\max\left(SI_{i}\times S_{SoC,i}\right)$$ (10)

Based on the above equation, BS expressing the best scenario, SI_i_ expressing the smoothness index (SI) value related to the i^th^ scenario and S_SoC,i_ is the slip value of the SoC index in the i^th^ scenario, which will be obtained according to (11).


$$S_{SoC,i}=\frac{100-BC\times SoCR_{i}}{100}$$ (11)

Whete BC is battery capacity and obtained from (6). The reason for multiplying the value by BC in the above equation is that with the increase in battery capacity, attention to battery life will be more important.

The value of SoCR_i_ is the range of SoC changes related to the i^th^ scenario, which will be obtained according to (12).


$$SoCR_{i}=\max\left(SoC_{i}\right)-\min\left(SoC_{i}\right)$$ (12)

In the above equation, SoC_i_ is the expression of SoS in the i^th^ scenario.

Based on this, the following scenarios are proposed for the operation of the battery storage:

- *Scenario 0*: WT without battery energy storage.
- *Scenario 1*: WT with battery energy storage by SoC variation range *from 0% to 100%*.
- *Scenario 2*: WT with battery energy storage by SoC variation range *from 10% to 90%*.
- *Scenario 3*: WT with battery energy storage by SoC variation range *from 20% to 80%*.
- *Scenario 4*: WT with battery energy storage by SoC variation range *from 30% to 70%*.
- *Scenario 5*: WT with battery energy storage by SoC variation range *from 0% to 50%*.
- *Scenario 6*: WT with battery energy storage by SoC variation range *from 50% to 100%*.

The reason for choosing these scenarios is to check different charging and discharging situations of the battery energy storage. By comparing the SI value in all these scenarios, it is possible to discuss the best operating situation of the battery to extend its life.

Moreover, the proposed approach relies on predefined SoC-based operating scenarios, eliminating the need for high-dimensional optimization algorithms. This enhances computational efficiency, making the methodology suitable for real-time applications. The derived decision metric, SI_i_ × S_SoCi_, serves as a lightweight yet effective trade-off indicator between smoothing performance and battery life expectancy. The algorithm’s simplicity enables practical implementation in embedded systems for microgrid operation.

## 4. Simulation results

To check the proposed procedure, simulation results from a wind turbine model 50 kW is used together with an induction generator. The specifications of wind turbine and induction generator are given in Tables 2 and 3, respectively [30].

**Table 2:** Wind turbine system specifications.

Rated power	50 [kW]
Rotor diameter	2/14 [m]
Air density	225/1 [kg/m3]
The highest value of the power factor	0.411
Optimum blade tip speed ratio	7/96
Low and High cut-off speed	3-25 [m/s]
Rated wind speed	12 [m/s]

**Table 3:** Induction generator specifications.

Rated power	50 [kW]
Stator voltage/frequency	380 [V]/60 [HZ]
Stator resistance	0.016 pu
Rotor resistance	0.015 pu
Stator leakage inductance	0.06 pu
Rotor leakage inductance	0.06 pu
Mutual inductance	5.3 pu

**Fig 4:**
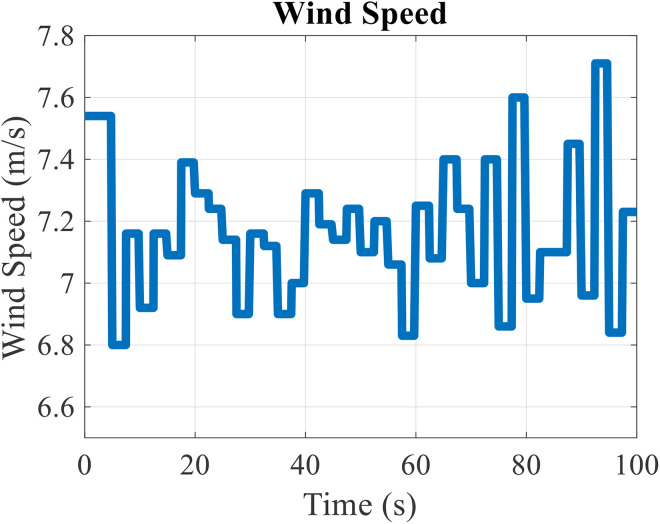
Wind speed applied to the wind turbine.

The value of wind speed during 100 seconds is shown in Fig 4. Each wind speed data is 2.5 seconds in duration, so 40 wind speed data will be obtained in 100 seconds [31]. The wind speed data in this study has changed between 6.8 meters per second and 7.8 m/s.

**Fig 5:**
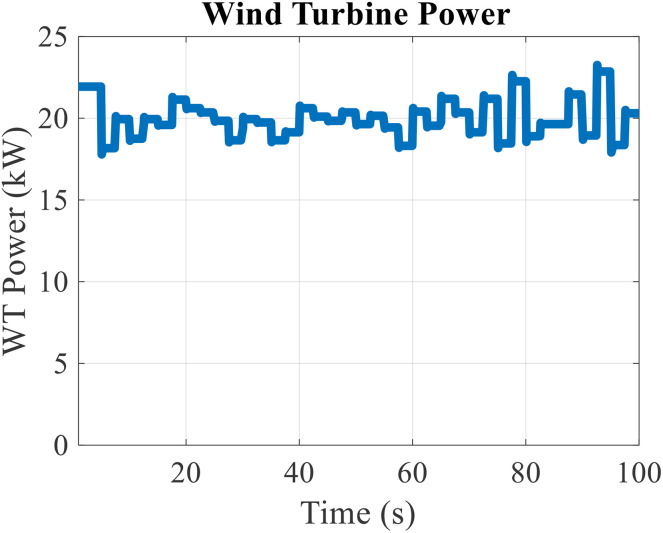
Wind turbine power output.

According to the wind speed data, the output power of the wind turbine will be as shown in Fig 5. According to this figure, it can be seen that when the wind speed increases, the output power of the wind turbine also increases, and on the contrary, when the wind speed decreases, the output power of the wind turbine will decrease. Therefore, it can be seen that the fluctuations of the output power of the wind turbine are proportional to the wind speed and have a high value. The maximum output power of the wind turbine is equal to 23.254 kW and the minimum value is 17.789 kW. In other words, the variation range of wind turbine output power is equal to 5.465 kW.

The entire simulation framework was developed using MATLAB/Simulink R2016-b. The simulation integrates a wind turbine model (aerodynamic and induction generator), a BESS, and a micro-grid structure. Key aspects include:

- **Turbine modeling:** Based on C_p_–λ curve and pitch angle characteristics.
- **Generator modeling:** Three-phase squirrel-cage induction machine.
- **Battery:** Modeled with SoC constraints and dynamic charge/discharge controller.
- **Control block:** Logic-based switching for BESS operation based on load-following.
- **Simulation horizon:** 100 seconds with a 0.1-second time step.
- **Scenarios:** Defined SoC range for each test case (Scenarios 1–6).
- **Evaluation Metrics:** RI, SI, and BEE as performance indicators.

This setup enables real-time interaction between wind power generation, storage control, and load response under variable wind conditions.

In the following, at first, the size of the battery used in the micro-grid and the corresponding wind turbine will be obtained, and then the production of battery storage power in each scenario will be investigated.

### 4.1. Battery capacity calculation

In this section, the battery capacity will be determined. To determine the required BESS capacity for a wind turbine with a power of 50 kW and a maximum demand of 20 kW for 10 seconds, it is necessary to follow the following steps:

- Calculating of the required energy in the 10-second peak demand:

Required~Energy=20(kw)×10(s)=200(kws)

- Converting to kilowatt-hours:

$$200\left(kws\right)=\frac{200}{3600}\approx 0.0556\left(kWh\right)$$

- Determining the DoD for the battery (For this calculation, we assume DOD as 50%).
- Calculating of the required BESS capacity (battery efficiency value of 95% is included):

$$\text{BC}=\frac{\text{0}\mathrm{.0556}}{0.5\times 0.95}≃0.117\left(kwh\right)$$

- To ensure design safety and commercial availability, a 0.15 kWh modular BESS would be appropriate for this smoothing interval.

Therefore, to handle a 10-second 20 kW peak, a BESS with a minimum capacity of approximately 0.12 kWh is required. Commercially, this can be satisfied with a 0.15 kWh unit or multiple modules thereof.

### 4.2. Scenarios analysis

**Fig 6:**
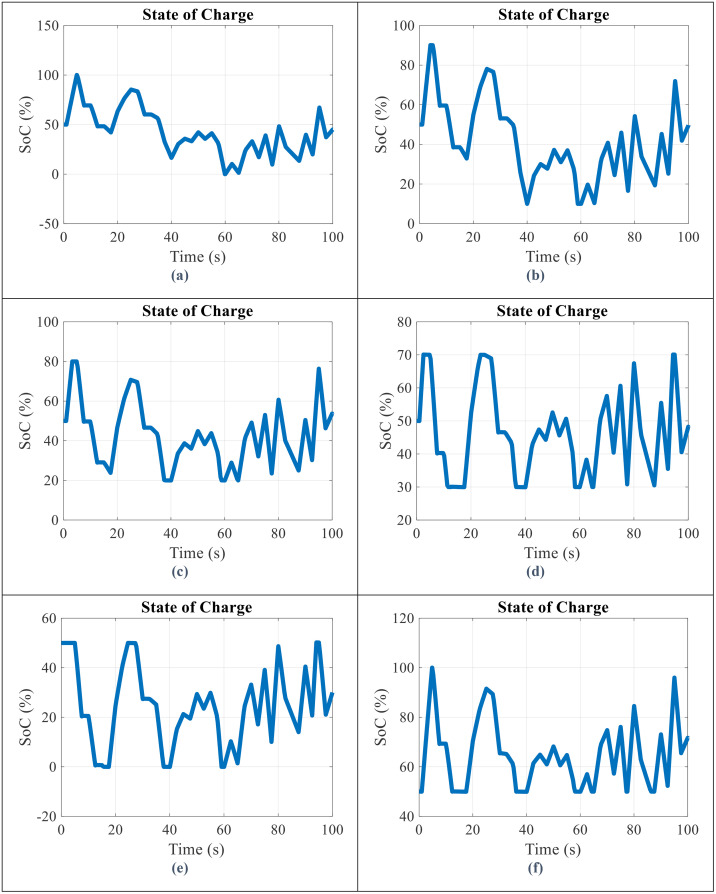
State of charge (SoC) for scenario (a) 1 (b) 2 (c) 3 (d) 4 (e) 5 (f) 6.

**Fig 7:**
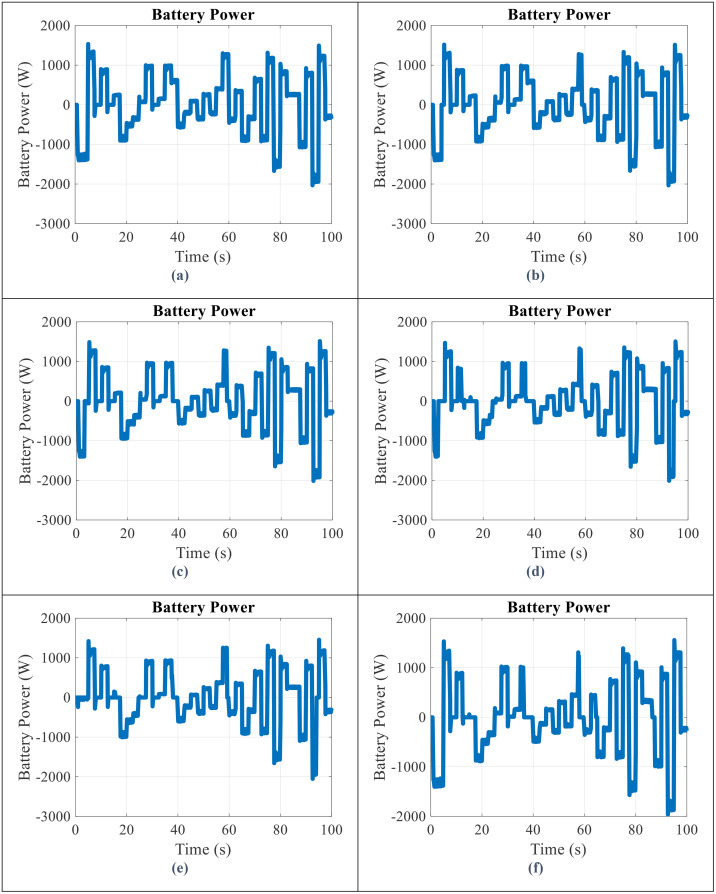
Battery power output for scenario (a) 1 (b) 2 (c) 3 (d) 4 (e) 5 (f) 6.

**Fig 8:**
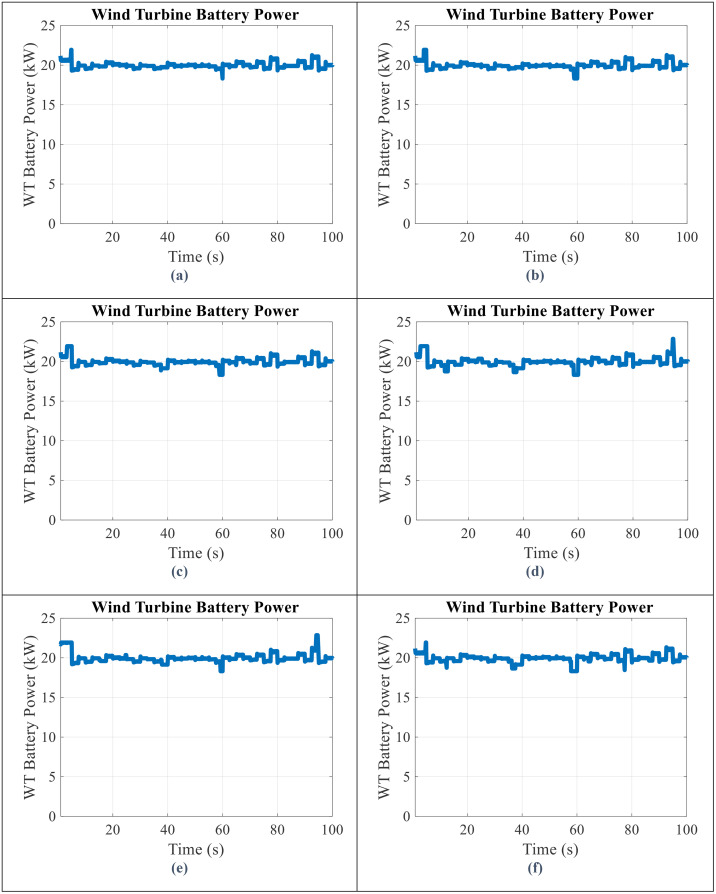
WT and Battery power output for scenario (a) 1 (b) 2 (c) 3 (d) 4 (e) 5 (f) 6.

By applying the battery storage in parallel with the wind turbine, the SoC of battery, the output power obtained from the battery storage, and the WT and battery power output in all scenarios will be obtained as shown in Fig 6–8, respectively.

Based on the data illustrated in Fig 6, the following observations can be made:

- SoC Range of each scenario is in each defined interval.
- When the battery has reached SoC range, the output power from the battery and WT will fluctuate.
- Reaching SoC range will lead to output power fluctuations, and the more these charging and discharging ranges are reached, the worse the condition of SI will be.

According to Fig 7:

- When the value of the battery storage power is positive, the battery is being discharged (flowing power from the battery to the micro-grid) and when the value is negative, the battery is being charged (flowing power from the micro-grid to the battery).
- This issue will be obtained by comparing the production power of the wind turbine and the load power (20 kW). In other words, when the power of the wind turbine is greater than the load power, the excess power is stored in the battery and leads to its charging, and on the contrary, when the power of the wind turbine is less than the load power, the lack of power is compensated by the battery and leads to discharge.
- The range of battery storage power changes is in the range of 20 kW.
- By reducing the SoC range, the amount of momentary fluctuations in charging and discharging the battery has increased. In other words, the lower SoC range, the more the battery will reach full charge/discharge state and the swing will have a faster moment.
- The amount of battery energy exchanged (BEE) in different scenarios is shown in Table 4. According to this table, the amount of energy exchanged in the state of upper SoC range is upper than other states. Accordingly, the energy exchanged of the battery in scenario 1 is more than the other scenarios.

**Table 4:** Battery energy exchange in all scenario.

Scenario No.	BEE (GWs)
1	618.88
2	593.72
3	561.83
4	501.51
5	513.19
6	541.93

According to Fig 8:

- The greater the SoC range, the lower the fluctuations in the output power of the WT and battery.
- The difference in scenarios 2–6 depends on the wind speed. In other words, if the production power of the WT is higher than 20 kW, the lower SoC ranges are more useful, and on the contrary, if the production power of the WT is less than 20 kW, the upper SoC range will be more useful.

## 5. Discussion

In this section, the results obtained in different scenarios will be discussed. The summary of the results in different scenarios is shown in Table 5. According to these results:

**Table 5:** Simulation results in all scenarios.

Parameter		*Scenario No.*						
		*0*	*1*	*2*	*3*	*4*	*5*	*6*
Battery Parameter	BC (kws)	0.421						
	SoC range	---	0-100	10-90	20-80	30-70	0-50	50-100
	SoCR_i_	---	100	80	60	40	50	50
	**S**_**SoC,i**_	**---**	**0.579**	**0.663**	**0.747**	**0.832**	**0.789**	**0.789**
Micro-Grid Parameter	P_ave_ (kW)	19.97						
	$\sum_{n=1}^{n_{spikes}}\left|P_{n}-P_{ave}\right|$ (kW)	17.78	6.05	6.05	6.31	8.84	9.27	7.40
	$\sum_{n=1}^{n_{valleys}}\left|P_{n}-P_{ave}\right|$ (kW)	17.02	3.64	4.91	5.81	7.86	6.84	7.94
	RI_i_ (kW)	34.79	9.70	10.96	12.12	16.70	16.11	15.34
	**SI**_**i**_ ** (%)**	**0**	**72.12**	**68.50**	**65.16**	**52.00**	**53.69**	**55.91**
Decision Making	SI_i_ × S_SoC,i_	0	41.75	45.43	48.7	43.24	42.39	44.14
	Scenario Rank	7	6	2	1	4	5	3
	BS				**✓**

- In the zero scenario, where the energy storage battery is not located, the highest output power fluctuations (RI = 34.79 kW fluctuations) were observed, and the SI = 0 in this scenario (without any smoothing).
- With the placement of the battery in the rest of the scenarios, the SI is opposite to zero, and in other words, the amount of fluctuations is less than the zero scenario (RI_i_ < RI_0_, i = scenario number, and i ≠ 0).
- The longer the SoC, the larger SI. in other words, the fluctuation reduction in output power will be greater by increasing the range of SoC changes.
- By comparing scenarios 5 and 6, it can be concluded that swing SoC is more practical in the above ranges and shows a larger SI value. Therefore, it can be said that the state of SoC > 50% in the proposed battery is more useful and (SI_i_ × S_SoC,i_) will increase as much as 2 units.
- The improved smoothing performance observed in Scenario 6 (SoC range 50–100%) compared to Scenario 5 (SoC range 0–50%) can be attributed to electrochemical behavior of the BESS. Batteries typically exhibit higher efficiency, lower impedance, and more stable voltage characteristics at higher states of charge. These properties allow the BESS to respond more effectively and rapidly to power fluctuations when operating in the upper SoC region. In contrast, the lower SoC region generally suffers from diminished dynamic performance, thereby limiting its smoothing capability.
- By comparing scenarios 1–4, it can be found that the SI distance in scenarios 1 and 2 and also in scenarios 2 and 3 is much lower than the SI distance in scenarios 3 and 4. Based on this, it can be said that scenario 4 is not cost-effective, but scenario 3 is a more suitable proposal. Because:
  - By limiting the SoC from the range (0-100) to the range (10-90), only less than 4% of the SI is reduced, which is not very significant.
  - By limiting the SoC from the range (10-90) to the range (20-80), only about 3% of the SI is reduced, which is not very significant.
  - But by limiting the SoC from the range (20-80) to the range (30-70), about 13% of the SI has been reduced, which is a significant amount.
- It can also be seen that scenarios 5 and 6 also have a much lower SI value compared to scenarios 1–3 and will not be a suitable proposal.
- The value of (SI_i_ × S_SoC,i_) in scenario 3 is the largest and it means that this scenario will be a more appropriate choice in this case study.
- Finally, it can be suggested that the scenario 3, which include small range of SoC (and will lead to a longer lifespan), has an acceptable SI value (SI = 65.16%), and the largest amount of (SI_i_ × S_SoC,i_), is the best suggestion would be to use a battery storage.
- Therefore, the SoC range of 20%–80% (scenario 3) represents a moderately constrained window that provides a well-balanced solution: it achieves effective wind power smoothing (SI = 65.16%) while also limiting the extent of battery cycling, which directly contributes to reducing battery wear and prolonging lifespan. This scenario offers the optimal trade-off between performance and durability among all evaluated cases.

Although the simulation in this paper focuses on a 100-second time window to demonstrate the dynamic performance of the BESS under fast wind fluctuations, the proposed method is fully extendable to long-term wind datasets (e.g., hourly or minute-level data over days, months, or a full year). Such an extension would allow for capturing seasonal variability and cumulative battery usage metrics, which are essential for real-world planning. Future studies can implement this method using actual wind data collected from turbine SCADA systems or meteorological sources to validate the smoothing index (SI) and battery usage under practical conditions.

To contextualize the proposed SoC strategy, Table 6 provides a benchmark comparison against selected SoC management and power smoothing methods from the literature. Unlike model-predictive or fuzzy-based controllers that require complex tuning or computational overhead, our scenario-based approach evaluates a finite set of predefined SoC ranges using analytically derived metrics. Moreover, methods based on inertia or supercapacitors [13,17] often lack long-duration balancing or lifecycle awareness. Our approach thus provides a balance between practical deployment, smoothing effectiveness, and battery degradation mitigation.

**Table 6:** Benchmark comparison of SoC/power smoothing approaches.

Method/ Reference	Control Strategy	Computational Complexity	Battery Lifetime	Real-Time Capability	Key Limitation
Proposed Method (This Paper)	SoC scenario- based	Low (Analytical)	Considered (via SoC range)	Suitable	Limited to offline scenario evaluation
[21] (Fuzzy + Supercapacitor)	Fuzzy-based smoothing controller	Medium	Not explicitly addressed	Partial	Requires fuzzy tuning, weak on lifecycle
[13] (Inertia-Based Smoothing)	Turbine inertia control	Low	Not applicable	Yes	No energy storage → limited smoothing
[26] (Wide SoC Range with Li-ion)	Full SoC operation (0–100%)	Low	High degradation risk	Yes	Accelerated battery aging
[17] (ANN + Capacitor)	ANN-based predictive control	High	Implicit	Dependent on training data	Black-box model; lacks interpretability

Building upon the findings of this study, several research directions are proposed for future investigation:

- Long-term simulation using real wind profiles over seasonal or yearly horizons to evaluate cumulative battery utilization and renewable integration effectiveness.
- Battery degradation modeling by incorporating empirical or semi-empirical aging models (e.g., based on rainflow counting, temperature effects, and cycle depth) to evaluate SoC strategy impact on battery lifespan.
- Development of real-time control algorithms for adaptive SoC range management under forecast-based or demand-responsive control schemes.
- Economic assessment including cost-benefit analysis of BESS deployment under different SoC strategies, battery technologies, and market participation models.
- BESS siting and sizing optimization in distribution networks, considering both technical constraints and energy market signals.
- Hybrid energy storage systems (e.g., battery + supercapacitor) evaluation to combine short-term smoothing with long-term energy balancing.

These directions aim to enhance the robustness, practicality, and scalability of the proposed methodology in real-world microgrid and utility-scale applications.

## 6. Conclusion

As mentioned, the output power fluctuations of the wind turbine are fast and therefore the battery storage system is facing serious damage. Because it has to be charged and discharged quickly and its lifespan is greatly reduced. In this paper, an attempt was made to provide a procedure to determine the type of operation of the battery storage system next to the wind turbine resource, based on which, in addition to improving and smoothing the output power, the range of SoC changes of the battery is such that its lifespan is also improved. According to the results, the battery condition was simulated in different scenarios and the best scenario was selected. The proposed method is completely based on mathematical values and is far from being expert in quality, so the obtained result will be accurate. It should also be noted that the purpose of this paper is planning the use of battery storage. Therefore this analysis will be used at the beginning of the design of the wind turbine and battery storage system. Based on the simulation outcomes, the best operating condition is achieved using a moderately constrained SoC range of 20%–80%. This range successfully balances the power smoothing objective with the goal of minimizing battery stress and aging, making it the recommended configuration for practical deployment.

## Supporting information

S1 File Sim_and data. (RAR)

## References

[1] Energy Information Administration EIA. International energy outlook 2005. http://www.eia.doe.gov/iea

[2] El-Fouly THM, El-Saadany EF. One day ahead prediction of wind speed using annual trends. IEEE; 2006.

[3] Watson SJ. Application of wind speed forecasting to the integration of wind energy into a large scale power system. IEE Proc Gener Transm Distrib. 1994;141(4):357. doi: 10.1049/ip-gtd:19941215

[4] Yang H, Zhang C, Li J, Zhu L, Zhou K. A Novel robust energy storage planning method for grids with wind power integration considering the impact of hurricanes. IEEE Trans Sustain Energy. 2025;16(2):1388–400. doi: 10.1109/tste.2025.3527448

[5] Howlader AM, Urasaki N, Yona A, Senjyu T, Saber AY. A review of output power smoothing methods for wind energy conversion systems. Renew Sustain Energy Rev. 2013;26:135–46. doi: 10.1016/j.rser.2013.05.028

[6] Guo Y, Yousefi A. Determining the appropriate size of the electrical energy storage system of an energy process based on a solid oxide fuel cell and wind turbine. J Energy Storage. 2021;44:103430. doi: 10.1016/j.est.2021.103430

[7] Yoo JI, Kang YC, Yang D, Kim K-H, Park J-W. Power smoothing of a variable-speed wind turbine generator based on a two-valued control gain. IEEE Trans Sustain Energy. 2020;11(4):2765–74. doi: 10.1109/tste.2020.2975061

[8] Jin H, Liu P, Li Z. Dynamic modeling and design of a hybrid compressed air energy storage and wind turbine system for wind power fluctuation reduction. Comput Chem Eng. 2019;122:59–65. doi: 10.1016/j.compchemeng.2018.05.023

[9] Qu L, Qiao W. Constant power control of DFIG wind turbines with supercapacitor energy storage. IEEE Trans Ind Applicat. 2011;47(1):359–67. doi: 10.1109/tia.2010.2090932

[10] Kadri A, Marzougui H, Aouiti A, Bacha F. Energy management and control strategy for a DFIG wind turbine/fuel cell hybrid system with super capacitor storage system. Energy. 2020;192:116518. doi: 10.1016/j.energy.2019.116518

[11] Nasiri M, Milimonfared J, Fathi SH. Modeling, analysis and comparison of TSR and OTC methods for MPPT and power smoothing in permanent magnet synchronous generator-based wind turbines. Energy Conv Manag. 2014;86:892–900. doi: 10.1016/j.enconman.2014.06.055

[12] Lyu X, Zhao J, Jia Y, Xu Z, Po Wong K. Coordinated control strategies of PMSG-based wind turbine for smoothing power fluctuations. IEEE Trans Power Syst. 2019;34(1):391–401. doi: 10.1109/tpwrs.2018.2866629

[13] Zhao X, Yan Z, Xue Y, Zhang X-P. Wind power smoothing by controlling the inertial energy of turbines with optimized energy yield. IEEE Access. 2017;5:23374–82. doi: 10.1109/access.2017.2757929

[14] Howlader AM, Senjyu T, Saber AY. An integrated power smoothing control for a grid-interactive wind farm considering wake effects. IEEE Syst J. 2015;9(3):954–65. doi: 10.1109/jsyst.2014.2374311

[15] Islam F, Al-Durra A, Muyeen SM. Smoothing of wind farm output by prediction and supervisory-control-unit-based FESS. IEEE Trans Sustain Energy. 2013;4(4):925–33. doi: 10.1109/tste.2013.2256944

[16] Senjyu T, Sakamoto R, Urasaki N, Funabashi T, Fujita H, Sekine H. Output power leveling of wind turbine generator for all operating regions by pitch angle control. IEEE Trans Energy Conv. 2006;21(2):467–75. doi: 10.1109/tec.2006.874253

[17] Muyeen SM, Hasanien HM, Tamura J. Reduction of frequency fluctuation for wind farm connected power systems by an adaptive artificial neural network controlled energy capacitor system. IET Renew Power Gener. 2012;6(4):226–35. doi: 10.1049/iet-rpg.2010.0126

[18] Rajesh P, Naveen C, Venkatesan AK, Shajin FH. An optimization technique for battery energy storage with wind turbine generator integration in unbalanced radial distribution network. J Energy Storage. 2021;43:103160. doi: 10.1016/j.est.2021.103160

[19] Kong J, Kim ST, Kang BO, Jung J. Determining the size of energy storage system to maximize the economic profit for photovoltaic and wind turbine generators in South Korea. Renew Sustain Energy Rev. 2019;116:109467. doi: 10.1016/j.rser.2019.109467

[20] Sun C, leto S. A novel joint bidding technique for fuel cell wind turbine photovoltaic storage unit and demand response considering prediction models analysis Effect’s. Int J Hydrogen Energy. 2020;45(11):6823–37. doi: 10.1016/j.ijhydene.2019.12.210

[21] de Carvalho WC, Bataglioli RP, Fernandes RAS, Coury DV. Fuzzy-based approach for power smoothing of a full-converter wind turbine generator using a supercapacitor energy storage. Electric Power Syst Res. 2020;184:106287. doi: 10.1016/j.epsr.2020.106287

[22] Aranizadeh A, Mirmozaffari M, Khalatabadi Farahani B. Maximizing wind turbine power generation through adaptive fuzzy logic control for optimal efficiency and performance. Wind. 2025;5(1):4. doi: 10.3390/wind5010004

[23] Ozbak M, Ghazizadeh-Ahsaee M, Ahrari M, Jahantigh M, Mirshekar S, Mirmozaffari M, et al. Improving power output wind turbine in micro-grids assisted virtual wind speed prediction. Sustain Operations Comput. 2024;5:119–30. doi: 10.1016/j.susoc.2024.06.004

[24] Aranizadeh A, Zaboli A, Asgari Gashteroodkhani O, Vahidi B. Wind turbine and ultra-capacitor harvested energy increasing in microgrid using wind speed forecasting. Eng Sci Technol Int J. 2019;22(5):1161–7. doi: 10.1016/j.jestch.2019.08.006

[25] Tabosa da Silva PL, Rosas PAC, Castro JFC, Marques D da C, Aquino RRB, Rissi GF, et al. Power smoothing strategy for wind generation based on fuzzy control strategy with battery energy storage system. Energies. 2023;16(16):6017. doi: 10.3390/en16166017

[26] Behabtu HA, Vafaeipour M, Kebede AA, Berecibar M, Van Mierlo J, Fante KA, et al. Smoothing intermittent output power in grid-connected doubly fed induction generator wind turbines with Li-Ion batteries. Energies. 2023;16(22):7637. doi: 10.3390/en16227637

[27] Aranizadeh A, Shahrtash SM, Gholami A. Comprehensive condition assessment of circuit breakers in a power network for maintenance scheduling. IET Generation Trans Dist. 2023;17(15):3463–76. doi: 10.1049/gtd2.12908

[28] Aranizadeh A, Shahrtash SM, Gholami A. Prioritizing CBs maintenance and identifying mandatory maintenance at higher priorities. Int Trans Electric Energy Syst. 2022;2022:1–14. doi: 10.1155/2022/5008166

[29] Aranizadeh A, Shad H, Vahidi B, Khorsandi A. A novel small-scale wind-turbine blade failure detection according to monitored-data. Results Eng. 2025;25:103809. doi: 10.1016/j.rineng.2024.103809

[30] GHRE P. 50 kW wind turbine information pack. http://www.ghrepower.co.uk/pdfs/50kw_info_pack.pdf

[31] Riahy GH, Abedi M. Short term wind speed forecasting for wind turbine applications using linear prediction method. Renew Energy. 2008;33(1):35–41. doi: 10.1016/j.renene.2007.01.014

